# Dysregulation of the Excitatory Renal Reflex in the Sympathetic Activation of Spontaneously Hypertensive Rat

**DOI:** 10.3389/fphys.2021.673950

**Published:** 2021-06-03

**Authors:** Chao Ye, Fen Zheng, Jing-Xiao Wang, Xiao-Li Wang, Qi Chen, Yue-Hua Li, Yu-Ming Kang, Guo-Qing Zhu

**Affiliations:** ^1^Key Laboratory of Targeted Intervention of Cardiovascular Disease, Collaborative Innovation Center of Translational Medicine for Cardiovascular Disease, Department of Physiology, Nanjing Medical University, Nanjing, China; ^2^Department of Pathophysiology, Nanjing Medical University, Nanjing, China; ^3^Department of Physiology and Pathophysiology, Cardiovascular Research Center, Xi’an Jiaotong University School of Medicine, Xi’an, China

**Keywords:** renal reflex, hypertension, sympathetic activity, blood pressure, kidney

## Abstract

Excessive sympathetic activation plays crucial roles in the pathogenesis of hypertension. Chemical stimulation of renal afferents increases the sympathetic activity and blood pressure in normal rats. This study investigated the excitatory renal reflex (ERR) in the development of hypertension in the spontaneously hypertensive rat (SHR). Experiments were performed in the Wistar-Kyoto rat (WKY) and SHR aged at 4, 12, and 24 weeks under anesthesia. Renal infusion of capsaicin was used to stimulate renal afferents, and thus, to induce ERR. The ERR was evaluated by the changes in the contralateral renal sympathetic nerve activity and mean arterial pressure. At the age of 4 weeks, the early stage with a slight or moderate hypertension, the ERR was more enhanced in SHR compared with WKY. The pressor response was greater than the sympathetic activation response in the SHR. At the age of 12 weeks, the development stage with severe hypertension, there was no significant difference in the ERR between the WKY and SHR. At the age of 24 weeks, the later stage of hypertension with long-term several hypertensions, the ERR was more attenuated in the SHR compared with the WKY. On the other hand, the pressor response to sympathetic activation due to the ERR was smaller at the age of 12 and 24 weeks than those at the age of 4 weeks. These results indicate that ERR is enhanced in the early stage of hypertension, and attenuated in the later stage of hypertension in the SHR. Abnormal ERR is involved in the sympathetic activation and the development of hypertension.

## Introduction

Excessive sympathetic activity is closely associated with hypertension, chronic heart failure, and chronic kidney disease (Chen et al., 2015; Grassi and Ram, 2016; Cheng et al., 2019). Most of the patients with chronic kidney diseases have an excessive sympathetic activation and hypertension, which is closely related to the increased morbidity and mortality of cardiovascular events (Kaur et al., 2017). Sympathetic overactivity is found not only in various hypertensive animal models including the spontaneously hypertensive rat (SHR) (Fan et al., 2012), obesity-related hypertensive rats (Xiong et al., 2012), renovascular hypertensive rats (Chen et al., 2011), and DOCA-sal hypertensive rats (Yemane et al., 2010), but also in patients with essential hypertension (Fisher and Fadel, 2010) and secondary hypertension (Vecchione et al., 2000; Lambert et al., 2007; Neumann et al., 2007). The excessive sympathetic activation plays a pathogenic role in the occurrence and development of hypertension and related organ damage (Grassi et al., 2015). Intervention of sympathetic overactivity is an important strategy for attenuating hypertension and its complication (Esler, 2014; Seravalle et al., 2014).

Kidney plays critical roles in the sympathetic activation in hypertension and chronic kidney diseases (Rettig et al., 1989, 1990; Iliescu et al., 2015). Renal nerves comprise of the afferent sensory fibers and efferent sympathetic fibers. The afferent sensory activity from the kidney to the brain is involved in regulating the sympathetic activity and blood pressure (Kopp, 2015; Bie and Evans, 2017; Milanez et al., 2020). Selective removal of renal afferent fibers reduces the blood pressure and sympathetic activity in a rat model of renovascular hypertension (Lopes et al., 2020). Recently, we have shown that the chemical stimulation of kidney in normal rats with capsaicin causes an excitatory renal reflex (ERR), which results in the sympathetic activation and pressor responses (Ye et al., 2020). The capsaicin-induced ERR is mediated by angiotensin II in the hypothalamic paraventricular nucleus (PVN), which acts on AT_1_R, and in turn activates NADPH oxidase, causing oxidative stress and the subsequent NFκB activation and IL-1β production in the PVN in normal rats (Qiu et al., 2020; Zheng et al., 2020). The renal afferent input increases the activity of some neurons in the PVN (Xu et al., 2015), and destruction of the PVN neurons abolishing the capsaicin-induced ERR (Ye et al., 2020).

In the recent years, catheter-based renal sympathetic denervation (RDN) is the most extensively investigated approach for intervention of hypertension by interrupting the activity of both afferent and efferent renal nerves (Lauder et al., 2021; Weber and Osborn, 2021). It is important to know the changes of the ERR in the pathogenesis of hypertension. Unfortunately, the changes of the ERR-induced by the chemical stimulation of renal afferents in hypertension are still unknown. SHR is the most widely used animal model of essential hypertension. The genetic hypertension model has many similarities with human essential hypertension in the pathophysiological progress, neuroendocrine changes, clinical course, and secondary diseases (Bell et al., 2004; Graham et al., 2005). In the present study, we investigated the changes of the ERR in the occurrence and development of hypertension in the SHR.

## Materials and Methods

### Animals

Male WKY and SHR at ages four, 12, and 24 weeks were obtained from the Vital River Laboratory Animal Technology Co., Ltd. (Beijing, China). The rats were kept in a temperature-controlled room on a 12-h cycle of light/darkness. Tap water and normal rat chow were available *ad libitum*. The experiments were approved by the Experimental Animal Care and Use Committee of Nanjing Medical University, and performed in accordance with the recommendations in the NIH guidelines (Eighth edition, 2011) listed in the Guide for the Care and Use of Laboratory Animals.

### General Procedures

Rats were anesthetized intraperitoneally with a mixture of urethane (800 mg/kg) and α-chloralose (40 mg/kg). Depth of anesthesia was assessed by both the paw withdrawal and corneal reflexes (Zhang et al., 2014). The rats were kept in a supine position, and a vertical incision was made in the middle of the neck to expose the trachea and carotid artery. Positive pressure ventilation *via* endotracheal intubation with room air was performed using a small animal ventilator (51,600, Stoelting, Chicago, IL, United States). A PE50 catheter was implanted into the right common carotid artery for blood pressure recording. Right and left kidneys were, respectively, exposed *via* flank incisions for preparing renal stimulation to induce ERR and renal sympathetic nerve activity (RSNA) recording. After the surgery, rats were allowed to stabilize for more than 30 min before intervention. Finally, the rats were euthanized by a rapid intravenous injection of pentobarbital sodium (100 mg/kg).

### Assessment of ERR

Our previous study has shown that the infusion of capsaicin into the cortico-medullary border of the kidney caused greater ERR effects than those in the cortex or medulla of the kidney. Infusion of capsaicin into the upper, lateral, or lower parts of the kidney showed similar ERR effects (Ye et al., 2020). Therefore, the cortico-medullary border of the lateral part of the kidney was selected for the infusion site in inducing ERR (Qiu et al., 2020; Zheng et al., 2020). Intravenous infusion of the same dose of capsaicin failed to cause significant effects on the RSNA, mean arterial pressure (MAP), and heart rate (HR), excluding the possibility that the effects of the renal infusion of capsaicin might be caused by leaking into the blood circulation (Ye et al., 2020). The right kidney was exposed through a flank incision. An outer diameter of 0.31 mm stainless steel tube was inserted horizontally from the lateral margin of the kidney to the hilum level of the kidney. The tip of the tube rested on the edge of the renal cortex and medulla, where the tube encountered a slight resistance. A PE50 catheter was connected to the tube with a PM2000B programmable pressure injector (MicroData Instrument, NJ, United States). The ERR was induced by a renal infusion of capsaicin (1 nmol/μL) at 1.0 μL/min for 20 min, and evaluated by the capsaicin-induced changes in the RSNA and MAP. The same amount of vehicle was used as a control. At the end of the experiment, the same volume of Evans Blue was infused for the histological identification of the infusion sites. Capsaicin was purchased from MedChemExpress (Monmouth Junction, NJ, United States).

### RSNA Recording

Renal sympathetic nerve activity was continuously recorded as we reported previously (Ye et al., 2020). Left renal sympathetic nerve was exposed through a left retroperitoneal incision in a prone position. The nerve was separated, and cut at the distal end to eliminate the renal afferent activity. The renal nerve was placed on a pair of platinum electrode, and soaked in paraffin oil at 37°C. Renal nerve signal was amplified by 4-Channel Differential Amplifier (Warner Instrument, Hamden, CT, United States) with a band-pass between 100 and 3,000 Hz. RSNA was integrated at a 100-ms time constant using the LabChart 8 software (ADInstruments). Background noise was recorded after cutting the central end of the renal nerve. RSNA data were obtained by subtracting the background noise. The percentage change in the integrated RSNA from the baseline value was calculated after renal infusion.

### Blood Pressure Recording

Before the acute experiment, blood pressure of tail artery was measured in a conscious state with a non-invasive computerized tail-cuff system (NIBP, ADInstruments). Rats were warmed at 28°C for 10–20 min so that the pulsation of the caudal artery could be detected to reach the pulse level. The systolic blood pressure (SBP) was determined by averaging 10 measurements. During the acute experiment, the right common carotid artery was exposed *via* a vertical incision in the middle of the neck. A PE50 catheter filled with normal saline containing heparin (50 IU/mL) was implanted into the right common carotid artery. Blood pressure was continuously recorded *via* a pressor transducer connected with the catheter using an 8SP PowerLab system with the data acquisition software (ADInstruments, Bella Vista, NSW, Australia).

### Statistics

Renal sympathetic nerve activity, MAP, and HR changes were determined by a one-minute average at the time frame of the maximal RSNA responses to the chemicals. All data were expressed as mean ± SE. Student’s *t*-test was used to compare the difference between two groups. Paired t test were used to compare the values before and after the intervention. One-way and two-way ANOVA followed by Bonferroni’s *post hoc* analysis were used for multiple comparisons. *P* < 0.05 was considered statistically significant.

## Results

### ERR in the WKY and SHR at the Age of 4 Weeks

**FIGURE 1:**
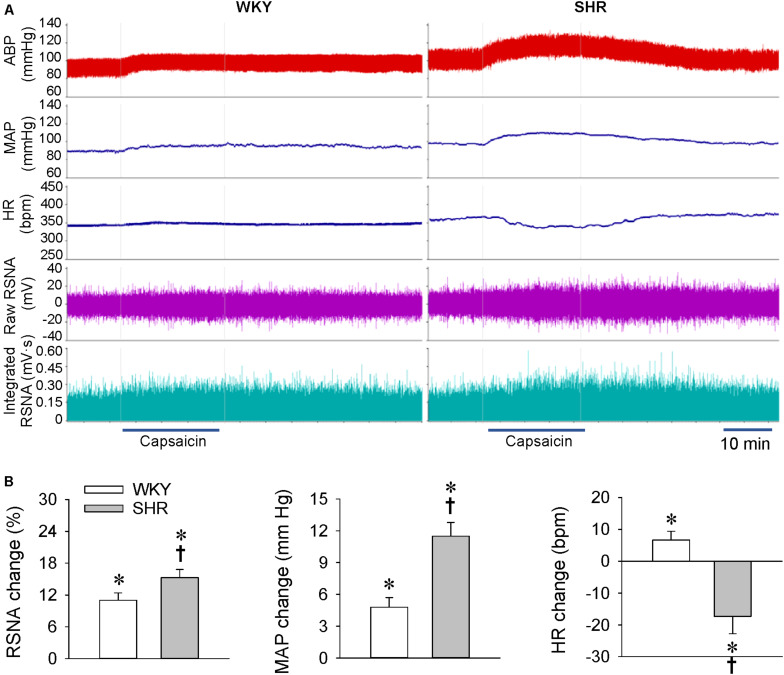
ERR in the WKY and SHR at the age of 4 weeks. The ERR was induced by renal infusion of capsaicin at 1 nmol/min for 20 min. **(A)** representative recordings showing the recordings of the capsaicin-induced ERR. **(B)** bar graph showing the capsaicin-induced ERR. Values are mean ± SE. ^∗^*P* < 0.05 vs the values before renal infusion; ^†^*P* < 0.05 vs WKY. *n* = 6.

Our previous study has shown that the renal infusion of capsaicin dose-dependently induces ERR in normal rats (Ye et al., 2020). The dose of capsaicin at 1 nmol/min for 20 min was selected to induce ERR, and the vehicle for renal infusion had no significant effects on the RSNA, MAP, and HR (Qiu et al., 2020; Ye et al., 2020; Zheng et al., 2020). At the age of 4 weeks, the early stage with slight or moderate hypertension, unilateral renal infusion of capsaicin caused immediate increases in the contralateral RSNA and MAP in both the WKY and SHR, lasting for at least 30 min. Capsaicin reached its maximal effect at about 15 min after the beginning of the renal infusion. However, capsaicin reduced the HR in SHR, which occurred a little later than the capsaicin-induced pressor response (Figure 1A). The ERR was significantly enhanced in the SHR compared with the WKY. However, capsaicin increased the HR in WKY, but decreased in SHR (Figure 1B).

### ERR in WKY and SHR at the Age of 12 Weeks

**FIGURE 2:**
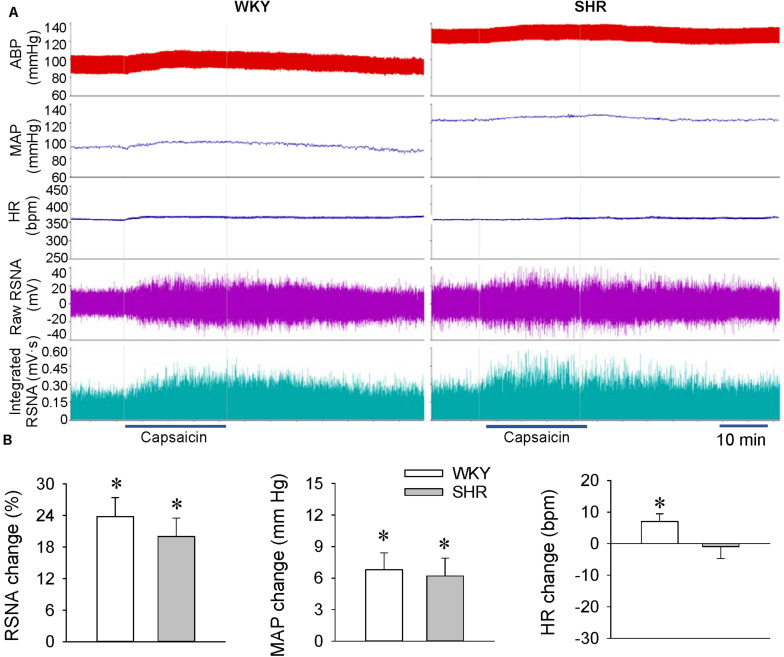
ERR in the WKY and SHR at the age of 12 weeks. The ERR was induced by renal infusion of capsaicin at 1 nmol/min for 20 min. **(A)** representative recordings showing the recordings of the capsaicin-induced ERR. **(B)** bar graph showing the capsaicin-induced ERR. Values are mean ± SE. ^∗^*P* < 0.05 vs the values before renal infusion. No significant difference was found between the WKY and SHR. *n* = 6.

Renal infusion of capsaicin caused a rapid increase in the RSNA and MAP in both the WKY and SHR at the age of 12 weeks, the development stage with severe hypertension (Figure 2A). Unexpectedly, the capsaicin-induced ERR was not significantly enhanced in the SHR compared with the WKY. Renal infusion of capsaicin increased the HR in WKY, but had no significant effects in SHR (Figure 2B).

### ERR in WKY and SHR at the Age of 24 Weeks

**FIGURE 3:**
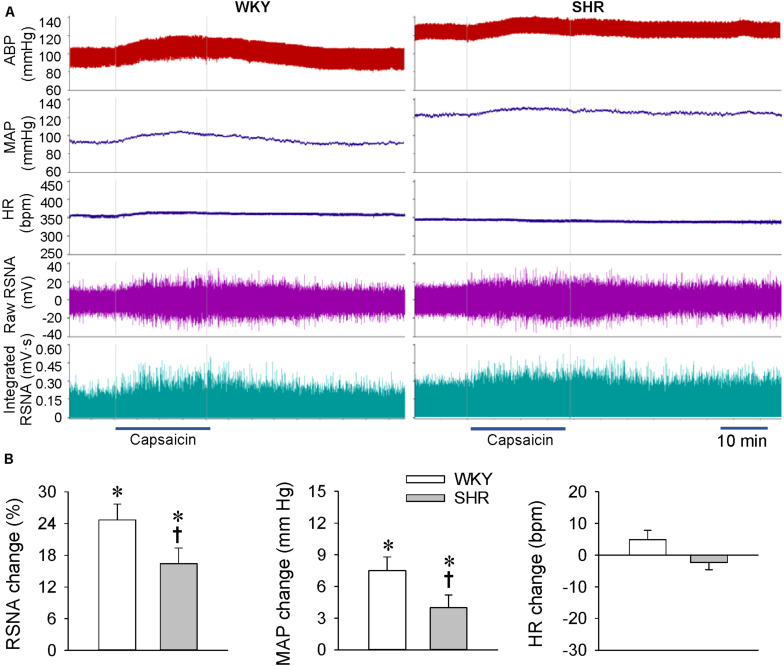
ERR in the WKY and SHR at the age of 24 weeks. The ERR was induced by renal infusion of capsaicin at 1 nmol/min for 20 min. **(A)** representative recordings showing the recordings of the capsaicin-induced ERR. **(B)** bar graph showing the capsaicin-induced ERR. Values are mean ± SE. ^∗^*P* < 0.05 vs the values before renal infusion; ^†^*P* < 0.05 vs WKY. *n* = 6.

At the age of 24 weeks, the later stage of hypertension with long-term several hypertensions, renal infusion of capsaicin increased the RSNA and MAP in both the WKY and SHR (Figure 3A). However, the capsaicin-induced ERR was significantly attenuated in the SHR compared with the WKY. Renal infusion of capsaicin had no significant effects on the HR in both the WKY and SHR (Figure 3B).

### Comparison of ERR at Different Ages of Hypertension in WKY and SHR

**FIGURE 4:**
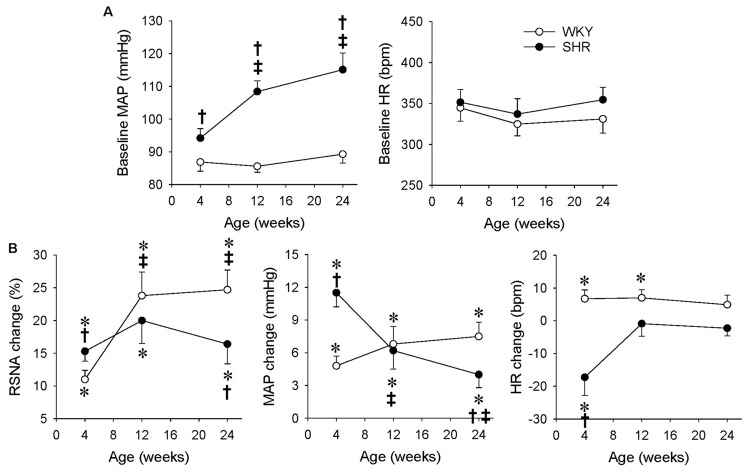
Comparison of ERR in the WKY and SHR at the ages of 4, 12, and 24 weeks. The ERR was induced by renal infusion of capsaicin at 1 nmol/min for 20 min. **(A)** Baseline MAP and HR. ^†^*P* < 0.05 vs WKY. ^‡^*P* < 0.05 vs 4 weeks. **(B)** capsaicin-induced ERR. Values are mean ± SE. ^∗^*P* < 0.05 vs the values before renal infusion; ^†^*P* < 0.05 vs WKY. ^‡^*P* < 0.05 vs 4 weeks. *n* = 6.

There was no significant difference in the body weight between the WKY and SHR at the ages of 4, 12, and 24 weeks (Table 1). The SBP of the SHR in a conscious state at the ages of 4, 12, and 24 weeks were significantly higher than those in the WKY, and the SBP in the SHR at the ages of 12 and 24 weeks were higher than that in the SHR at the age of 4 weeks (Table 1). Similarly, the baseline MAP of the SHR under an anesthetic at the ages of 4, 12, and 24 weeks were significantly higher than those in the WKY, and the baseline MAP in the SHR at the ages of 12 and 24 weeks was higher than that in the SHR at the age of 4 weeks (Figure 4A). In order to better understand the changes of ERR in the different periods of hypertension, we presented the results mentioned above in another format (Figure 4B). The RSNA response to capsaicin in WKY at the ages of 12 and 24 weeks was more enhanced than that in the WKY at the age of 4 weeks, while the MAP response to capsaicin in the SHR at the ages of 12 and 24 weeks was more attenuated than that in the SHR at the age of 4 weeks. Capsaicin-induced reduction in HR only occurred in the SHR at the age of 4 weeks (Figure 4B).

**TABLE 1:** Body weight and systolic blood pressure measured in a conscious state.

**Age (weeks)**	**BW (g)**		**SBP (mm Hg)**	
	**WKY**	**SHR**	**WKY**	**SHR**
4	99.7 ± 4.6	102.5 ± 5.6	118.0 ± 3.8	143.5 ± 5.7*
12	249.0 ± 8.2^†^	244.0 ± 7.9^†^	121.3 ± 3.9	182.2 ± 8.6*^†^
24	389.3 ± 7.5^†‡^	368.7 ± 9.8^†‡^	122.5 ± 4.9	191.0 ± 6.3*^†^

Values are mean ± SE. *P < 0.05 vs WKY. ^†^*P* < 0.05 vs four weeks. ^‡^*P* < 0.05 vs 12 weeks. *n* = 6. BW, body weight; SBP, systolic blood pressure.

### Difference in the RSNA and MAP Responses to Capsaicin in the WKY and SHR

**FIGURE 5:**
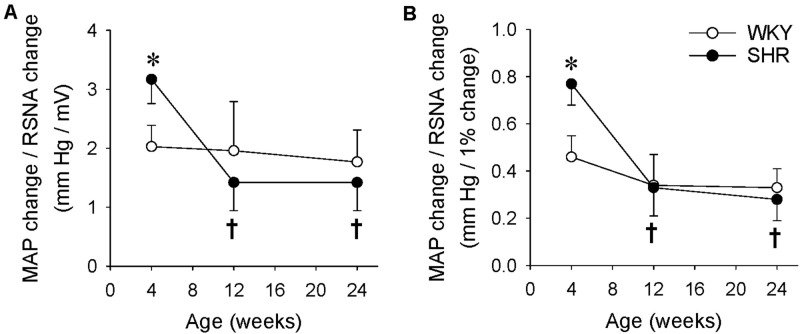
Difference of the RSNA and MAP responses to renal infusion of capsaicin in the WKY and SHR at the ages of 4, 12, and 24 weeks. The ERR was induced by renal infusion of capsaicin at 1 nmol/min for 20 min. **(A)** The ratio of MAP change to RSNA change induced by capsaicin. The values were expressed as mmHg/mV. **(B)** The ratio of MAP change to RSNA change induced by capsaicin. The values were expressed as mm Hg/1% RSNA change. Values are mean ± SE. ^∗^*P* < 0.05 vs WKY. ^†^*P* < 0.05 vs 4 weeks. *n* = 6.

We further compared the difference in the RSNA and MAP responses to capsaicin with the ratio of the MAP change to the integrated RSNA change (P/A index), an index to evaluate the relationship of MAP and RSNA. The P/A index was calculated as the ratio of MAP change in mmHg to the integrated RSNA change in mV (Figure 5A) or to the integrated RSNA change in percentage (Figure 5B). The P/A index was greater in the SHR than that in the WKY at the age of 4 weeks, but there was no significant difference in the P/A index between the WKY and SHR at the age of 12 or 24 weeks. The upregulated P/A index in the SHR at the age of 4 weeks was reduced to the normal level at the ages of 12 and 24 weeks (Figures 5A,B).

## Discussion

Renal infusion of capsaicin to induce ERR dose-dependently increased the contralateral RSNA and MAP in normal rats. The ERR can be induced by renal infusion of several chemicals such as capsaicin, angiotensin II, bradykinin, and adenosine (Ye et al., 2020). Capsaicin stimulates afferents and increases the afferent nerve activity at a low concentration, but causes denervation at a high concentration (Fitzgerald, 1983). The concentration of capsaicin in the present study was used to stimulate the renal afferents and cause ERR (Qiu et al., 2020; Ye et al., 2020; Zheng et al., 2020), which was much lower than the concentration used for denervation in previous studies (Fitzgerald, 1983; Foss et al., 2015). The capsaicin-induced ERR existed in the SHR at the ages of four, 12, and 24 weeks, indicating that the ERR is involved in the sympathetic activation and hypertension from the early stage to the sustained stage of hypertension in SHR. It is noted that the ERR was enhanced in the SHR aged at 4 weeks compared with the WKY, indicating that the enhanced ERR is crucial for the sympathetic activation and the occurrence and development of hypertension in the early stage. The findings suggest the importance of early intervention of the enhanced ERR in retarding and attenuating sympathetic activation and hypertension. On the other hand, vascular remodeling contributes to the development and complications of hypertension (Schiffrin, 2012). Intervention of sympathetic activation or a variety of major targets in arteries at the early stage of hypertension not only attenuates hypertension, but also vascular remodeling in the young SHR (Fan et al., 2012; Sun et al., 2017; Ren et al., 2020). We speculate that early intervention of the enhanced ERR may have beneficial effects in attenuating vascular remodeling in hypertension. Although the ERR was attenuated in the 24-weeks-old SHR compared with the WKY, the attenuated ERR was still an important factor to increase the sympathetic activity and blood pressure in a sustained hypertension.

An interesting finding in the present study was that the increase in RSNA was smaller but the increase in blood pressure was greater in the 4-week old SHR than those in the WKY. We propose to use the P/A index to reflect the relationship between the RSNA and MAP changes. The increased P/A index means that smaller changes in the renal sympathetic nerve activity (A) cause greater changes in the blood pressure (P). In this study, the P/A index in the young SHR was much greater than that in the young WKY or adult SHR, suggesting that an increased RSNA is more important for hypertension in the young SHR. In the WKY rats, capsaicin-induced the sympathetic activation increasing the HR. However, renal infusion of capsaicin in the four-weeks-old SHR reduced the HR, which may be secondary to the enhanced baroreceptor reflex because the capsaicin-induced HR reduction response appeared a little later than the pressor response. In the SHR at the ages of 12 and 24 weeks, renal infusion of capsaicin had no significant effects on the HR. The possible explanation may be that the role of sympathetic activation in increasing the HR is attenuated by the role of the baroreceptor reflex in reducing the HR, and/or the parasympathetic control of HR is dampened in the adult SHR.

Transient receptor potential vanilloid 1 (TRPV1) is a non-selective cation channel, which is primarily expressed in the sensory Aδ- and C-fibers and primary sensory neurons. The TRPV1 can be activated by capsaicin, endovanilloids, and a variety of chemical and physical stimuli such as lipid metabolites, acidic pH, and noxious heat (Zhong et al., 2019). Capsaicin, a selective TRPV1 agonist, increases the ipsilateral afferent renal nerve activity in the Wide-type mice but not in the TRPV1 knockout mice (Zhong et al., 2019). Pretreatment with a TRPV1 competitive antagonist capsazepine abolishes the capsaicin-induced ERR (Qiu et al., 2020). These findings indicate that the renal infusion of capsaicin-induced ERR is mediated by the TRPV1 receptors in the kidney.

Renal stimulation induces two opposite sympathetic responses, the excitatory renal reflex (ERR) and inhibitory renal reflex (IRR), which may be related to the site of stimulation, types of stimuli, and pathophysiological state (Kopp, 2015). ERR can be induced by the renal infusion of capsaicin, bradykinin, adenosine, and angiotensin II at the cortico-medullary border of the kidney, which increases the sympathetic activity and blood pressure (Ye et al., 2020). IRR can be induced by increasing the ureteral pressure or retrograde ureteropelvic perfusion with 0.9 M NaCl, which increases the ipsilateral afferent renal nerve activity but decreases the contralateral efferent renal nerve activity in normal WKY rats, but not in SHR (Kopp et al., 1987). Activation of renal afferents by the increased renal pelvic pressure, bradykinin, prostaglandin E2, substance P, and norepinephrine exerts an inhibitory effect on RSNA to minimize sodium retention (Kopp, 2015). The function of sensory fibers containing TRPV1 is impaired in obesity, diabetes, and aging (Zhong et al., 2019). TRPV1 expression and function in renal sensory fibers were impaired in the Dahl salt-sensitive hypertensive rats fed with a high-salt diet (Li and Wang, 2008). In the present study, the capsaicin-induced ERR was attenuated in the SHR at the age of 24 weeks compared with the WKY, which might be attributed to the impaired function of sensory fibers containing TRPV1. A limitation is that we did not measure the TRPV1 receptor expression in the kidneys between the WKY and SHR rats, which needs further investigation.

Excessive sympathetic activity plays crucial roles in the pathogeneses of hypertension, chronic heart failure, and chronic kidney disease (Fitzgerald, 1983; Chen et al., 2015; Grassi and Ram, 2016; Cheng et al., 2019). Renal afferent activity may contribute to the sympathetic overactivity in these diseases (Esler, 2014; Frame et al., 2016; Kaur et al., 2017). Catheter-based radiofrequency renal denervation (RFRD) was used as a therapy for hypertension, chronic kidney disease, and chronic heart failure (Veelken and Schmieder, 2014; Frame et al., 2016; Carlstrom, 2017). However, the renal denervation therapy destroys both the afferent and efferent renal nerves. The present study shows the important roles of renal afferent activity in the sympathetic activation in hypertension, especially in the early stage of hypertension. Selectively renal afferent denervation or efferent denervation to interrupt ERR may have its unique advantage in the treatment of hypertension.

In conclusion, abnormal ERR is involved in the sympathetic activation and development of hypertension. ERR is enhanced in the early stage of hypertension, and is attenuated in the later stage of severe hypertension in SHR. ERR-induced sympathetic activation is associated with a stronger pressor response in the early stage of hypertension.

## Data Availability Statement

The original contributions presented in the study are included in the article/supplementary material, further inquiries can be directed to the corresponding author.

## Ethics Statement

The animal study was reviewed and approved by Experimental Animal Care and Use Committee, Nanjing Medical University.

## Author Contributions

CY, QC, Y-HL, Y-MK, and G-QZ conceptualized and designed the study. CY, FZ, J-XW, and X-LW performed the research. CY, FZ, and G-QZ analyzed the data and contributed to the methods or models. CY and G-QZ wrote the original draft. All authors have read and agreed to the published version of the manuscript.

## Conflict of Interest

The authors declare that the research was conducted in the absence of any commercial or financial relationships that could be construed as a potential conflict of interest.

## References

[B1] Bell D. Kelso E. J. Argent C. C. Lee G. R. Allen A. R. McDermott B. J. (2004). Temporal characteristics of cardiomyocyte hypertrophy in the spontaneously hypertensive rat. *Cardiovasc. Pathol.* 13 71–78. 10.1016/s1054-8807(03)00135-2 15033155

[B2] Bie P. Evans R. G. (2017). Normotension, hypertension and body fluid regulation: brain and kidney. *Acta Physiol.* 219 288–304. 10.1111/apha.12718 27214656

[B3] Carlstrom M. (2017). Therapeutic value of renal denervation in cardiovascular disease? *Acta Physiol.* 220 11–13. 10.1111/apha.12816 28421719

[B4] Chen A. D. Zhang S. J. Yuan N. Xu Y. De W. Gao X. Y. (2011). AT_1_ receptors in paraventricular nucleus contribute to sympathetic activation and enhanced cardiac sympathetic afferent reflex in renovascular hypertensive rats. *Exp. Physiol.* 96 94–103. 10.1113/expphysiol.2010.054353 21097645

[B5] Chen W. W. Xiong X. Q. Chen Q. Li Y. H. Kang Y. M. Zhu G. Q. (2015). Cardiac sympathetic afferent reflex and its implications for sympathetic activation in chronic heart failure and hypertension. *Acta Physiol.* 213 778–794. 10.1111/apha.12447 25598170

[B6] Cheng Z. J. Wang R. Chen Q. H. (2019). Autonomic rregulation of the cardiovascular system: diseases, treatments, and novel approaches. *Neurosci. Bull.* 35 1–3. 10.1007/s12264-019-00337-0 30659525 PMC6357280

[B7] Esler M. (2014). Sympathetic nervous system moves toward center stage in cardiovascular medicine: from Thomas Willis to resistant hypertension. *Hypertension* 63 e25–32. 10.1161/HYPERTENSIONAHA.113.02439 24420544

[B8] Fan Z. D. Zhang L. Shi Z. Gan X. B. Gao X. Y. Zhu G. Q. (2012). Artificial microRNA interference targeting AT1a receptors in paraventricular nucleus attenuates hypertension in rats. *Gene Ther.* 19 810–817. 10.1038/gt.2011.145 21956687

[B9] Fisher J. P. Fadel P. J. (2010). Therapeutic strategies for targeting excessive central sympathetic activation in human hypertension. *Exp. Physiol.* 95 572–580. 10.1113/expphysiol.2009.047332 20304932 PMC2858400

[B10] Fitzgerald M. (1983). Capsaicin and sensory neurones–a review. *Pain* 15 109–130. 10.1016/0304-3959(83)90012-x 6189047

[B11] Foss J. D. Wainford R. D. Engeland W. C. Fink G. D. Osborn J. W. (2015). A novel method of selective ablation of afferent renal nerves by periaxonal application of capsaicin. *Am. J. Physiol Regul. Integr. Comp. Physiol.* 308 R112–R122. 10.1152/ajpregu.00427.2014 25411365 PMC4297859

[B12] Frame A. A. Carmichael C. Y. Wainford R. D. (2016). Renal afferents. *Curr. Hypertens. Rep.* 18:69. 27595156 10.1007/s11906-016-0676-z PMC5011151

[B13] Graham D. McBride M. W. Brain N. J. Dominiczak A. F. (2005). Congenic/consomic models of hypertension. *Methods Mol. Med.* 108 3–15. 10.1385/1-59259-850-1:003 16028672

[B14] Grassi G. Ram V. S. (2016). Evidence for a critical role of the sympathetic nervous system in hypertension. *J. Am. Soc. Hypertens.* 10 457–466. 10.1016/j.jash.2016.02.015 27052349

[B15] Grassi G. Mark A. Esler M. (2015). The sympathetic nervous system alterations in human hypertension. *Circ. Res.* 116 976–990. 10.1161/circresaha.116.303604 25767284 PMC4367954

[B16] Iliescu R. Lohmeier T. E. Tudorancea I. Laffin L. Bakris G. L. (2015). Renal denervation for the treatment of resistant hypertension: review and clinical perspective. *Am. J. Physiol. Renal Physiol.* 309 F583–94. 10.1152/ajprenal.00246.2015 26224718 PMC4593817

[B17] Kaur J. Young B. E. Fadel P. J. (2017). Sympathetic overactivity in chronic kidney disease: consequences and mechanisms. *Int. J. Mol. Sci.* 18:E1682. 10.3390/ijms18081682 28767097 PMC5578072

[B18] Kopp U. C. (2015). Role of renal sensory nerves in physiological and pathophysiological conditions. *Am. J. Physiol. Regul. Integr. Comp. Physiol.* 308 R79–95. 10.1152/ajpregu.00351.2014 25411364 PMC4297860

[B19] Kopp U. C. Smith L. A. DiBona G. F. (1987). Impaired renorenal reflexes in spontaneously hypertensive rats. *Hypertension* 9 69–75. 10.1161/01.hyp.9.1.69 3793202

[B20] Lambert E. Straznicky N. Schlaich M. Esler M. Dawood T. Hotchkin E. (2007). Differing pattern of sympathoexcitation in normal-weight and obesity-related hypertension. *Hypertension* 50 862–868. 10.1161/hypertensionaha.107.094649 17909120

[B21] Lauder L. Bohm M. Mahfoud F. (2021). The current status of renal denervation for the treatment of arterial hypertension. *Prog. Cardiovasc. Dis.* Online ahead of print. 10.1016/j.pcad.2021.02.005 33587963

[B22] Li J. Wang D. H. (2008). Role of TRPV1 channels in renal haemodynamics and function in Dahl salt-sensitive hypertensive rats. *Exp. Physiol.* 93 945–953. 10.1113/expphysiol.2008.042036 18403445 PMC2693710

[B23] Lopes N. R. Milanez M. I. O. Martins B. S. Veiga A. C. Ferreira G. R. Gomes G. N. (2020). Afferent innervation of the ischemic kidney contributes to renal dysfunction in renovascular hypertensive rats. *Pflugers Arch.* 472 325–334. 10.1007/s00424-019-02346-4 31925527

[B24] Milanez M. I. O. Veiga A. C. Martins B. S. Pontes R. B. Bergamaschi C. T. Campos R. R. (2020). Renal sensory activity regulates the γ-aminobutyric acidergic inputs to the paraventricular nucleus of the hypothalamus in Goldblatt hypertension. *Front. Physiol.* 11:601237. 10.3389/fphys.2020.601237 33384613 PMC7769809

[B25] Neumann J. Ligtenberg G. Klein I. H. Boer P. Oey P. L. Koomans H. A. (2007). Sympathetic hyperactivity in hypertensive chronic kidney disease patients is reduced during standard treatment. *Hypertension* 49 506–510. 10.1161/01.hyp.0000256530.39695.a3 17224471

[B26] Qiu Y. Zheng F. Ye C. Chen A. D. Wang J. J. Chen Q. (2020). Angiotensin type 1 receptors and superoxide anion production in hypothalamic paraventricular nucleus contribute to capsaicin-induced excitatory renal reflex and sympathetic activation. *Neurosci. Bull.* 36 463–474. 10.1007/s12264-019-00460-y 31989424 PMC7186294

[B27] Ren X. S. Tong Y. Qiu Y. Ye C. Wu N. Xiong X. Q. (2020). MiR155-5p in adventitial fibroblasts-derived extracellular vesicles inhibits vascular smooth muscle cell proliferation via suppressing angiotensin-converting enzyme expression. *J. Extracell. Vesicles* 9:1698795. 10.1080/20013078.2019.1698795 31839907 PMC6896498

[B28] Rettig R. Folberth C. Stauss H. Kopf D. Waldherr R. Unger T. (1990). Role of the kidney in primary hypertension: a renal transplantation study in rats. *Am. J. Physiol.* 258 F606–11. 10.1152/ajprenal.1990.258.3.F606 2138422

[B29] Rettig R. Stauss H. Folberth C. Ganten D. Waldherr B. Unger T. (1989). Hypertension transmitted by kidneys from stroke-prone spontaneously hypertensive rats. *Am. J. Physiol.* 257 F197–F203. 10.1152/ajprenal.1989.257.2.F197 2669526

[B30] Schiffrin E. L. (2012). Vascular remodeling in hypertension: mechanisms and treatment. *Hypertension* 59 367–374. 10.1161/hypertensionaha.111.187021 22203749

[B31] Seravalle G. Mancia G. Grassi G. (2014). Role of the sympathetic nervous system in hypertension and hypertension-related cardiovascular disease. *High Blood Press. Cardiovasc. Prev.* 21 89–105. 10.1007/s40292-014-0056-1 24789091

[B32] Sun H. J. Ren X. S. Xiong X. Q. Chen Y. Z. Zhao M. X. Wang J. J. (2017). NLRP3 inflammasome activation contributes to VSMC phenotypic transformation and proliferation in hypertension. *Cell Death. Dis.* 8:e3074. 10.1038/cddis.2017.470 28981106 PMC5680591

[B33] Vecchione C. Argenziano L. Fratta L. Pompeo F. Trimarco B. (2000). Sympathetic nervous system and hypertension in diabetic patients. *Diabetes Nutr. Metab.* 13 327–331. 11232757

[B34] Veelken R. Schmieder R. E. (2014). Renal denervation–implications for chronic kidney disease. *Nat. Rev. Nephrol.* 10 305–313. 10.1038/nrneph.2014.59 24733118

[B35] Weber M. A. Osborn J. W. (2021). Improved understanding of renal nerve anatomy: an opportunity to enhance denervation treatment of hypertension. *JACC Cardiovasc. Interv.* 14 316–318. 10.1016/j.jcin.2020.11.003 33541542

[B36] Xiong X. Q. Chen W. W. Han Y. Zhou Y. B. Zhang F. Gao X. Y. (2012). Enhanced adipose afferent reflex contributes to sympathetic activation in diet-induced obesity hypertension. *Hypertension* 60 1280–1286. 10.1161/hypertensionaha.112.198002 23033372

[B37] Xu B. Zheng H. Liu X. Patel K. P. (2015). Activation of afferent renal nerves modulates RVLM-projecting PVN neurons. *Am. J. Physiol. Heart Circ. Physiol.* 308 H1103–H1111. 10.1152/ajpheart.00862.2014 25637549 PMC4551125

[B38] Ye C. Qiu Y. Zhang F. Chen A. D. Zhou H. Wang J. J. (2020). Chemical stimulation of renal tissue induces sympathetic activation and pressor response via hypothalamic paraventricular nucleus. *Neurosci. Bull.* 36 143–152. 10.1007/s12264-019-00417-1 31392556 PMC6977808

[B39] YemaneH.BusauskasM.BurrisS. K.KnuepferM. M. (2010). Neurohumoral mechanisms in deoxycorticosterone acetate (DOCA)-salt hypertension in rats. *Exp. Physiol.* 95 51–55. 10.1113/expphysiol.2008.046334 19700514

[B40] Zhang L. L. Ding L. Zhang F. Gao R. Chen Q. Li Y. H. (2014). Salusin-beta in rostral ventrolateral medulla increases sympathetic outflow and blood pressure via superoxide anions in hypertensive rats. *J. Hypertens.* 32 1059–1067. 10.1097/hjh.0000000000000143 24621806

[B41] Zheng F. Ye C. Wan G. W. Zhou B. Tong Y. Lei J. Z. (2020). Interleukin-1β in hypothalamic paraventricular nucleus mediates excitatory renal reflex. *Pflugers Arch.* 472 1577–1586. 10.1007/s00424-020-02461-7 32915316

[B42] Zhong B. Ma S. Wang D. H. (2019). Ablation of TRPV1 elevates nocturnal blood pressure in Western diet-fed mice. *Curr. Hypertens. Rev.* 15 144–153. 10.2174/1573402114666181031141840 30381083 PMC6635649

